# Elderly Resistance vs. Youthful Acceptance: A Study on Insect Consumption across Age Groups

**DOI:** 10.3390/foods13162641

**Published:** 2024-08-22

**Authors:** María José Castro-Alija, Ghazal Zolfaghari, Carla Gutierrez Fernandez, Carlos Álvarez, Luis Carlos Ramón-Carreira, José María Jiménez, Irene Albertos

**Affiliations:** 1 Multidisciplinary Assessment and Intervention in Health Care and Sustainable Lifestyles, Recognized Research Group, University of Valladolid, 47003 Valladolid, Spain; mariajose.castro@uva.es (M.J.C.-A.); ghazal.zolfaghari23@estudiantes.uva.es (G.Z.); luiscarlos.ramon@uva.es (L.C.R.-C.); jose.maria.jimenez@uva.es (J.M.J.); 2 Department of Nursing, Faculty of Nursing, University of Valladolid, 47003 Valladolid, Spain; carlagutierrezfer@gmail.com; 3 Teagasc-Irish Agriculture and Food Development Authority, D15 DY05 Dublin, Ireland; carlos.alvarez@teagasc.ie

**Keywords:** entomophagy, insects, new foods, protein, sustainability, food neophobia

## Abstract

Insects have recently received much attention as sustainable protein sources due to their nutritional value and eco-friendliness. Unlike conventional livestock, insects require minimal resources and produce fewer greenhouse gas emissions. Moreover, insects offer high-quality protein, essential amino acids, and various vitamins and minerals. This study in Spain, specifically in Castilla y León, investigated insect consumption acceptance across age groups, particularly among older individuals, shedding light on factors influencing adoption. The findings inform strategies to address global protein deficiencies and advocate sustainable food practices, with implications for broader European research amidst challenges like water scarcity. **Methods:** A survey-based research approach collected data on attitudes, preferences, and motivations regarding insect consumption. Statistical analyses were conducted to identify demographic trends and significant associations. **Results**: Elderly participants expressed reluctance towards insect consumption but showed openness in survival scenarios. Younger individuals exhibited greater willingness to try insects, influenced by factors such as education and previous experiences. **Conclusion:** Understanding demographic variations in attitudes towards entomophagy is vital for fostering its acceptance. This study demonstrated that older individuals exhibit greater resistance to incorporating insects into their diets compared with younger individuals. Recommended strategies include incorporating insects discreetly into familiar foods and highlighting their nutritional advantages. Collaboration between researchers and stakeholders is essential for harnessing the potential of insects as a sustainable protein source.

## 1. Introduction

The expected global population increase to 9.7 billion by 2050, as projected by the United Nations [1,2], is likely to lead to a worldwide nutritional crisis. Meeting the growing demands of this expanding population requires increasing current food production, particularly through alternative protein sources to prevent resource depletion. Entomophagy, the consumption of insects as food, has emerged as a promising solution, especially in Europe [1,2,3,4].

Conventional livestock production has a significant environmental impact due to emissions of atmospheric pollutants [3]. In contrast, entomophagy offers a more environmentally friendly option, requiring less land and water for sustainability [3,4,5]. For instance, insect production has a superior feed conversion ratio (FCR), with crickets requiring only 1.7 kg of feed to produce 1 kg of biomass compared with 10 kg for 1 kg of beef [6,7].

Insect production also results in significantly fewer emissions of greenhouse gases compared with conventional livestock farming. Specifically, the production of insect protein releases 13 kgCO_2_ eq/kg, while beef protein releases 200 kgCO_2_ eq/kg [8].

As a result, future animal protein production will continue to require more land, potentially leading to de-forestation [6]. There are several arguments in favor of entomophagy and other similar novelty innovations as protein sources. Insects provide benefits to the environment and offer nutritive value, have short life cycles, high reproduction rates, and different collection processes [5]. For instance, crickets are capable of laying 1200 to 1500 eggs in thirty days, showcasing their reproductive abilities [7]. Additionally, insects can consume organic waste streams and serve as a form of bio-conversion, playing a role in waste management and nutrient cycling [8,9,10,11].

Entomophagy is also economical as the technology needed to produce insects is not complicated; this makes it suitable at different levels of socioeconomic developments, with the additional prospect of increasing efficiency through the usage of new technologies [7]. Insects are less pain-sensitive compared with livestock, reducing animal suffering, and they also have lower risk in transmitting zoonotic diseases [7].

The potential of insects for human consumption is explained by the amount of protein and other nutrients they contain. The literature has pointed out that insects are a source of high-biological-value protein, vitamins, and minerals [12,13]. They provide a comprehensive array of essential amino acids and can contain up to 70% lipids with a favorable fatty acid profile [2,14]. Insects also offer water-soluble vitamins like thiamine (B1), riboflavin (B2), and vitamin B12, as well as fat-soluble vitamins such as vitamin E (tocopherol) and A (retinol) [2,14]. They are a good source of dietary fiber and various minerals like iron, potassium, sodium, and calcium [14]. Due to their iron content, insects could help combat iron deficiency anemia and address zinc deficiencies in maternal and child populations [7].

Insects are beneficial in that they are low in calories, offering the possibility of reducing chronic diseases caused by cardiovascular disorders or obesity [5]. They can be integrated into hypocaloric diets for individuals with these conditions. In this way, consuming insects may have positive effects on human health by influencing the composition and balance of gut microbiota, as well as modulating certain blood parameters, revealing their antioxidant properties [9].

Insect consumption is versatile in culinary applications, including in raw, roasted, fried, or preserved food. They can be added to food products, milled into flour, or be the main ingredient in a dish. Insects have the potential to replace traditional high-energy fat sources [15].

Social factors such as culture, knowledge, awareness, and marketing strategies impact consumers’ attitudes towards entomophagy [16,17]. Sociocultural factors and legal frameworks also play a role in consumers’ acceptance of these products [18,19]. The European Union (EU), for example, emphasizes the importance of refining regulations to facilitate market energy and build consumer confidence [20]. Therefore, it is safe to say that technical and safety aspects are crucial for the continued growth of entomophagy consumption. The advancement of insect technology as a food source and adherence to safety standards directly influence consumer confidence and market expansion [21].

Various research papers have investigated different aspects of entomophagy acceptance across relevant stakeholders and through educational approaches. For instance, one study focused on how farmer acceptance influenced insect-based animal feeds [22], while another looked at consumer preference trends [23]. Studying educational programs and their effectiveness in shaping children’s perceptions of edible insects can be useful in improving entomophagy acceptance through education [24].

In the EU, insect consumption is classified as “novel food” according to Regulation 2015/2283 of the European parliament. Currently, the European Union permits the use of three insect species (*Tenebrio molitor*, *Locusta migratoria*, and *Acheta domesticus*) as supplementary ingredients in other foods or as snacks. Thorough evaluations are conducted to assess the potential allergenicity and toxicity risks of product before it is approved for market release [5].

The last but important factor that hinders the inclusion of insects in the diets practiced in Western countries is consumer attitude. Some reasons include cibophobia (fear of novel foods) and sociocultural perception regarding this resistance [3,4,5]. Despite this, the previous use of insects as a delicacy in Eastern cultures might one day become accepted in the West [9]. Thus, sociodemographic factors such as gender, age, and prior knowledge of the societies where insects are consumed also affect the likelihood of acceptance [4].

Nutrition plays an important role in health and disease prevention, and protein deficiency can have numerous negative effects, such as malnutrition and stunted growth in children. Finding new environmentally friendly sources of protein such as insects presents a viable solution to protein deficiencies and related diseases [1,5,9,24]. This research aims to provide valuable information on formulating solutions for potential future protein deficiencies, especially among the elderly in Castilla y León, distinguishing it from others in the field.

According to statistics reported by the National Statistics Institute (INE), the population of Castilla y León is decreasing in proportion to its area due to a decrease in the birth rate in this province. Traditionally, the population is aging, with a notable increase in the proportion of elderly people (aged 65 years and older) and a decrease in the proportion of younger people (aged 0–14 years) [25]. However, current research shows a lack of information regarding the attitudes of older adults towards entomophagy. This gap highlights the novelty and relevance of the present study, which focuses on this demographic group and their acceptance of insect-based foods.

This study particularly evaluates the likelihood of elderly people accepting insects as food, as insects can provide a source of protein and other nutrients. The findings will demonstrate the importance of incorporating this protein source into the diets of the elderly population in Castilla y León to combat potential protein deficiencies in the future, ultimately leading to more sustainable food choices for this group.

Given the expected increase in the world’s population by 2050 and the resulting strain on protein resources, the search for sustainable protein sources is crucial. The objective of this research is to provide valuable information to help address future protein shortages and develop solutions for a sustainable food supply.

## 2. Materials and Methods

### 2.1. Participants

This investigation recruited a sample population of 459 individuals from a specific geographical region, Castilla y León, Spain, with a focus on the Valladolid province. The sample size was calculated with a 95% confidence level, a 5% estimation error, and an additional 10% account for potential lack of interest in participating in the study. The participant demographics exhibited a sex distribution of 65.4% females and 34.6% males and a relatively even age distribution ranging from 18 to over 76 years old. A random selection process was used to ensure unbiased participant recruitment. The research adhered to a legal framework (Organic Law 3/2018) that protects data privacy (anonymity), participant autonomy (voluntariness), and ethical data use for specific academic purposes. Informed consent, in accordance with established ethical principles (Declaration of Helsinki), was obtained through information sheets and consent forms. Participation was contingent upon understanding the study’s objectives.

### 2.2. Research Design

This research utilized a descriptive, cross-sectional, and prospective design, conducted over a four-month period. However, it is worth mentioning that this study has not been validated. Ethical approval was granted by the designated institutional review board (Faculty of Nursing’s Ethics Committee at the University of Valladolid, Spain). All participants signed an informed consent form for inclusion in the study and use of clinical data for this research project (I.D. 2003/JRT2, approval date 23 February 2023). The work was conducted in accordance with the Code of Ethics of the World Medical Association (Declaration of Helsinki).

### 2.3. Materials and Data Collection

The research utilized computational resources for survey design, data entry, and subsequent analysis. Software applications such as Google Forms and statistical software like SPSS facilitated data management and statistical computations. Additionally, culturally adapted survey instruments (*n* = 120) were distributed to collect data.

The sample size was calculated with a 95% confidence level, a 5% estimation error, and an additional 10% account for potential lack of interest in participating in the study.

The initial phase involved conducting a comprehensive literature review within scientific publications to establish the research foundation. A survey instrument was developed, initially encompassing 15 questions. Through a refinement process, the final survey comprised 11 questions addressing participant demographics and entomophagy acceptance.

An online platform like Google Forms facilitated efficient survey administration and broader participant reach. For individuals lacking internet access, printed surveys were manually distributed. Completed surveys were then transferred to computers for data entry.

### 2.4. Variables and Measurement

The survey instrument explored various topics:

- Entomophagy acceptance: this dimension assessed participants’ willingness to include insects in their diet, encompassing both consumption of livestock raised on insect-based feed and acceptance of food products containing undisclosed insect ingredients.
- Demographic characteristics: age, sex, marital status, educational attainment, monthly income, nationality, residence, professional background, and dietary preferences were measured.
- Travel frequency: this variable assessed the frequency of international travel for business or leisure purposes.
- Food neophobia: the survey measured participant openness to trying new foods and trying foods from diverse cultures.
- Entomophagy knowledge and experience: this dimension assessed the participant´s awareness of insect consumption practices and any prior experiences with eating insects.
- Motivations for insect consumption: the survey explored the factors influencing participant´s willingness to incorporate insects into their diet, such as environmental benefits, nutritional properties, and personal curiosity.

### 2.5. Data Analysis

The data analysis utilized statistical software Statistical Package for the Social Sciences (SPSS, IBM, Armonk, NY, USA), version 28.0. Numerical variables were summarized using mean and standard deviation, while qualitative variables were analyzed using percentages. To determine sample size, confidence intervals were calculated at a 95% level. Variables with multiple response options were categorized numerically. Correlations between variables were assessed using the chi-square test or contingency coefficient, with statistical significance being set at a *p*-value of less than 0.05.

## 3. Results

The age distribution within the sample showed relative homogeneity, with each age group accounting for approximately 25% of the total population. It is worth noting that individuals over 76 years of age made up 6.1% of the sample, as shown in Table 1.

An analysis of participants’ marital status revealed a bimodal distribution, with approximately 45% categorized as married and a similar proportion classified as single. The remaining participants were categorized as widowed or separated.

Regarding educational attainment, nearly 60% of the sample possessed a baccalaureate or postgraduate degree, reflecting a high level of education. Secondary and primary school education were the next most prevalent categories, followed by a minority (3%) holding doctoral degrees.

**Figure 1:**
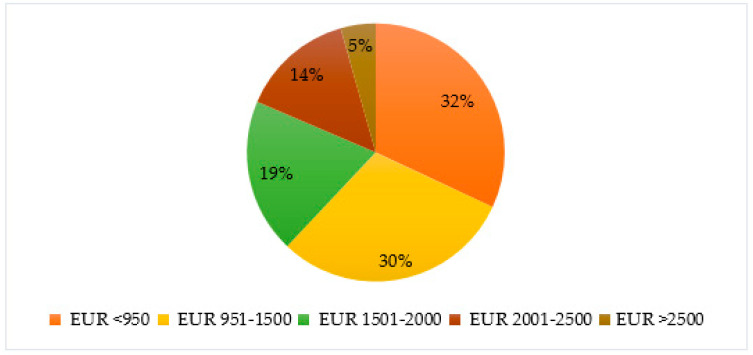
Graphic representation of the participants according to their monthly income in Euros (EUR).

The distribution of monthly income demonstrated a skew towards lower income brackets. The largest group comprised individuals earning less than EUR 950 per month, followed by the EUR 950–1500 income bracket. The remaining income categories exhibited similar representation, with a notable exception of individuals earning more than EUR 2500, who constituted a minority, as illustrated in Figure 1.

The sample showed a clear predominance of Spanish citizenship compared to participants from other countries. However, residential distribution demonstrated relative parity, with 57% residing in urban areas and 43% in rural settings. Notably, a majority of rural respondents originated from the Valladolid province. The participants encompassed a diverse range of professional backgrounds.

Dietary preferences showed significant diversity, with 72% being classified as omnivores. Meat and fish consumption were the second most common category, while other groups followed more plant-based diets. In terms of international travel frequency, approximately 35% reported no travel or traveling every two or more years. The remaining participants were evenly spread across two other frequency categories.

The results from the initial survey on the willingness to consume animals fed with insect-based feed showed that 44.4% of respondents agreed, with 29.4% strongly agreeing. A statistically significant correlation (*p <* 0.000) was found between this acceptance and age group, with individuals under 30 years showing the highest tolerance. Additionally, a difference was noted between participants with higher education levels and those with lower academic qualifications (*p* < 0.000). Furthermore, a significant correlation (*p* < 0.013) was discovered between respondents who reported making one trip or fewer annually and their readiness to consume traditional livestock raised on insect feed.

Participants were asked about their willingness to consume food products that contain hidden insect ingredients, like donuts, as a way to incorporate insects into their diet. The results showed that 46.8% of participants rejected this idea. Interestingly, education level had a significant influenced impact on this variable (*p* < 0.047), as respondents with doctoral degrees were more likely to accept such foods.

To explore potential preferences for insect-based food products available in the Spanish market, participants were asked to select their preferred options. Bakery products emerged as the most favored choice at 39.2%, followed by breakfast cereals at 30.7%, while sugary drinks were the least favored at 12.4%. Sex differences were statistically significant (*p* < 0.003), particularly in relation to alcoholic beverages and pre-cooked foods (*p* < 0.003), with males displaying greater willingness to consume such products. Age groups also exhibited variations in preferences, with young people showing higher receptivity towards alcoholic beverages (*p* < 0.001), sugary drinks (*p* < 0.017), and pre-cooked foods (*p* < 0.041). Educational level influenced preferences for pre-cooked meals (*p* < 0.026), with participants holding higher degrees expressing greater interest. Additionally, canned goods were more accepted by respondents who reported making one trip annually compared with those who traveled more frequently (*p* < 0.042).

**Figure 2:**
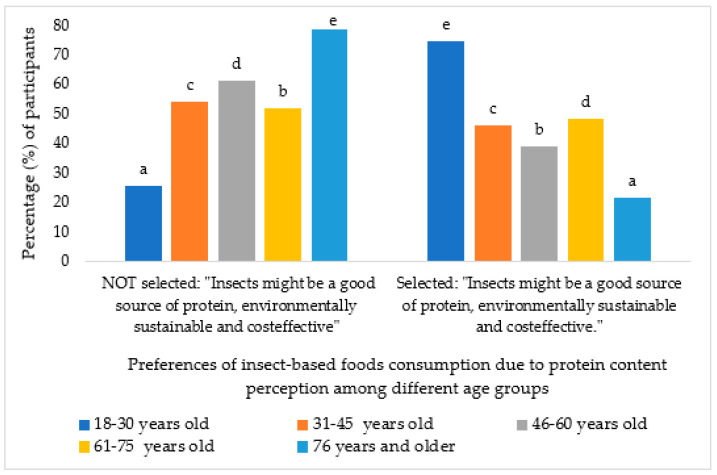
Percentage of participants who chose the option “For the protein contribution” in question 4 according to age groups. Different letters mean significant differences (*p* < 0.05).

Finally, respondents were asked about their perceptions regarding the emergence of insect consumption in Spain. The majority attributed it to the high protein content provided by insects (50.8%), followed by considerations of greater sustainability and cost-effectiveness (45.8%). Significant relationships were found between age groups: young people seem more determined that it is driven by the protein content (*p* < 0.000), as shown in Figure 2. 

**Figure 3:**
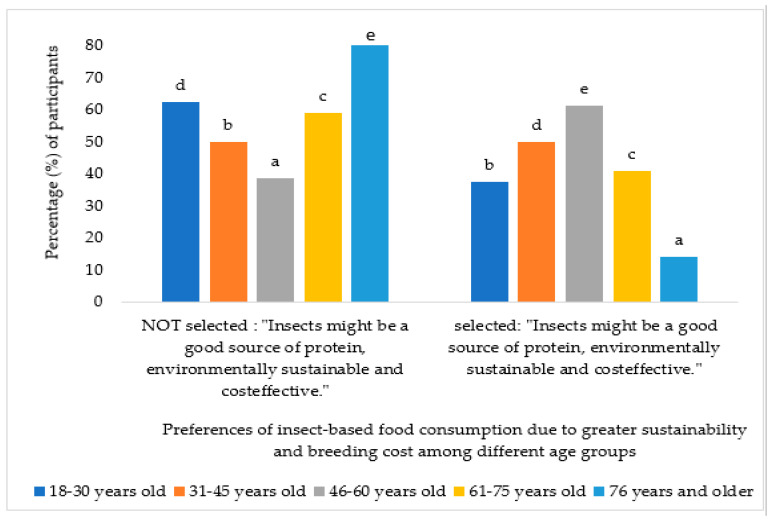
Percentage of participants who chose the option “For greater sustainability” in question 4 according to age groups. Different letters mean significant differences (*p* < 0.05).

The analysis revealed a statistically significant association (*p* < 0.000) between age and the underlying motivations for accepting entomophagy. Specifically, the 46–60-year-old age group demonstrated a preference for insect-based food products driven by sustainability concerns and the potential for reduced costs, as illustrated in Figure 3.

**Figure 4:**
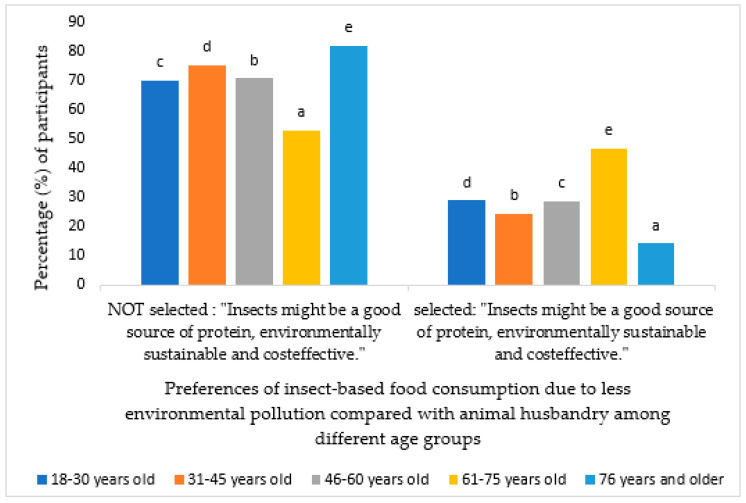
Percentage of participants who chose the option “For less environmental pollution” in question 4 according to age groups. Different letters mean significant differences (*p* < 0.05).

On the other hand, as shown in Figure 4, individuals aged 61 to 75 years believe that the recent concern for insects in Spain is a result of environmental pollution (*p* < 0.006). 

**Figure 5:**
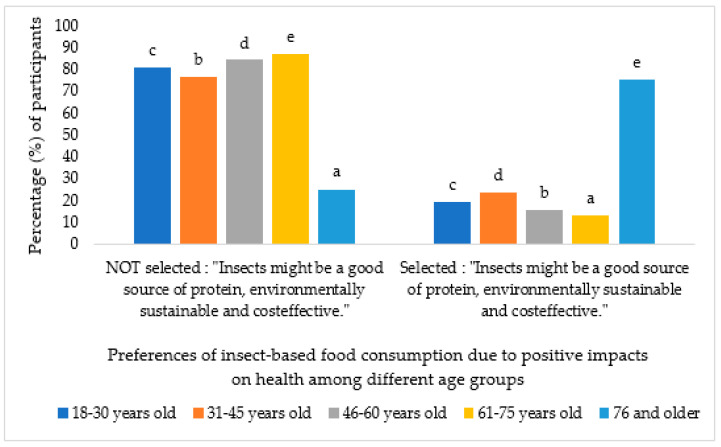
Percentage of participants who chose the option “For a positive impact on health” in question 4 according to age groups. Different letters mean significant differences (*p* < 0.05).

And finally, the older individuals believe that we are starting to discuss about this topic. because it can have a positive impact on health (*p* < 0.000) as shown in Figure 5.

A statistically significant association (*p* < 0.03) was observed between educational attainment and the primary motivations for accepting entomophagy. Individuals holding doctoral degrees exhibited the strongest preference for insect-based foods, driven by protein content, followed by those with bachelor’s degrees or secondary education. Notably, primary education level displayed a stronger correlation with the perceived positive health impact of entomophagy (*p* < 0.000). Additionally, a significant correlation was found with overseas travel frequency. Individuals traveling once a year highlighted potential environmental benefits like reduced pollution and lower costs (*p* < 0.024) associated with entomophagy. Furthermore, those who traveled annually reported a stronger association with the perceived positive health impact of insect consumption (*p* < 0.045), followed by non-travelers.

An analysis of motivations for routine insect consumption revealed that a substantial majority of participants (56.9%) would be willing to consume insects in a survival scenario. However, a noteworthy portion (24.4%) indicated an absolute aversion to insect consumption under any circumstances. Significant sex differences were observed, with women displaying a greater tendency to consider insect consumption due to potential environmental and sustainability benefits (*p* < 0.015). Age also played a role, with individuals under 30 years being more inclined to consume insects out of curiosity (*p* < 0.011) or indirectly as ingredients (*p* < 0.047), while those over 76 were primarily motivated by the perceived nutritional properties of insects (*p* < 0.001).

In terms of consumption methods, the majority (55.6%) preferred ingesting insects as an ingredient in other foods. A significant portion (31.2%) opted not to consume them at all, and a minority (7.4%) would consider consuming whole insects as a main dish.

Regarding knowledge about entomophagy, slightly more than half (50.8%) were aware of insect consumption practices in other cultures, followed by 45.3% who knew about different methods of insect preparation and consumption.

To assess the willingness of the Castilla y León population to include insects in their diet, participants’ food neophobia, or aversion to novel foods, was evaluated. Significant associations were found between willingness to try new foods, age, and international travel (*p* < 0.000), particularly among those aged 18 to 30 years. Additionally, a strong correlation was observed between food neophobia and willingness to consume insects, with significant results across all options (*p* < 0.000), indicating that many individuals hesitant to try new foods were also unwilling to consume insects.

Individuals who were unwilling to try foods from different cultures (*p* < 0.000) or hesitant to try new foods even with familiar company (*p* < 0.000) were similarly disinclined to try insects. Additionally, 44.4% of those with specific dietary preferences displayed resistance to incorporating insects into their diet (*p* < 0.001).

The understanding of participants regarding the availability of insects as a supermarket commodity in Spain was investigated. The majority (42.3%) assumed limited accessibility within the country, and an additional 22.7% were uncertain about its availability.

**Figure 6:**
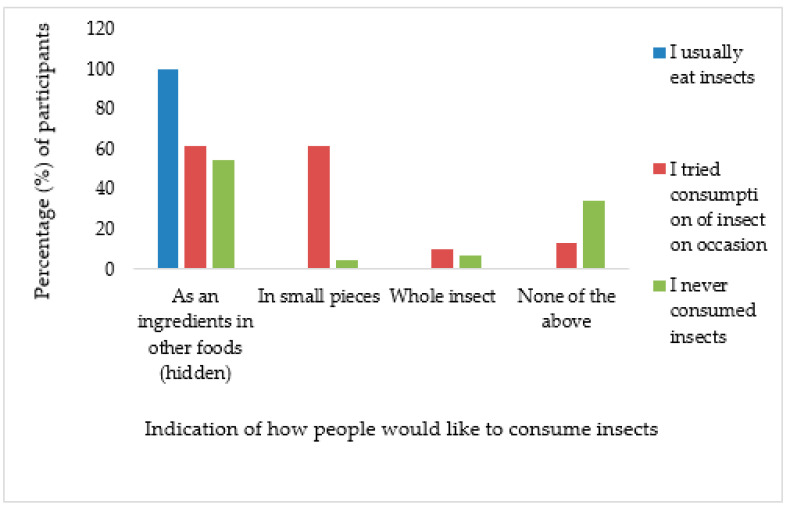
Percentage of participants who chose the hidden or no insect option based on their previous experience with insects.

Exploring the impact of prior experiences with insect consumption on the acceptance of a diet incorporating insects revealed that a vast majority (86.3%) of respondents had never tasted insects, while only 13.5% had experimented with them at some point. A significant association (*p* < 0.001), depicted in Figure 6, emerged between prior insect consumption experiences and willingness to integrate them into the diet, indicating that those with previous exposure to insects were more inclined to incorporate them as ingredients, in pieces or as a whole.

In the final survey, participants were presented with four statements about their willingness to consider entomophagy under different conditions. The idea of eating insects for their potential environmental benefits received the most diverse response, with complete agreement being the least common option. Statistical differences were noted among age groups (*p* < 0.012), with individuals over 61 years old showing more openness to this idea.

On the other hand, there was widespread agreement in considering entomophagy if insects were hidden in food products and offered health benefits. Similarly, there was a general consensus on turning to insect-based diets as a possible solution to global population growth.

Lastly, almost everyone indicated a willingness to consume animals that were fed insect-based food.

## 4. Discussion

The high popularity of entomophagy at this time can be attributed to the fact that insects possess value when consumed. Numerous studies have reinforced that they are rich sources of essential nutrients such as amino acids, vitamins, minerals, and bioactive compounds that have health benefits [14,26]. These attributes make insects a solution for the world’s malnutrition problems and a way to promote more sustainable food systems [27,28].

In integrated consumer psychology, defined as the field of study that examines the level of consumer acceptance of innovative foods, consumer psychology is relevant in the consumption of insect-based foods. As previously stated, there are key factors that affect consumers’ willingness to adopt these products into their diets, including cibophobia (reluctance to consume novel foods), disgust sensitivity, cultural backgrounds, and past experiences with entomophagy [15,29,30,31,32,33].

However, some individuals may be willing to taste foods that incorporate insects, while others may have strong cultural or perceived disgust towards insect-based foods [29,32]. 

The acceptance level of entomophagy is relative to cultural and demographic differences within a population. Studies show that these differences are influenced by cultural norms, eating habits, religion, and social status [9,16,34]. However, some countries have shown higher levels of acceptance of entomophagy, while others have developed cibophobia, leading to a cultural rejection of insects as foods [9,16,34].

Other research conducted in different countries, including non-EU and culturally distant countries, also shows an increasing interest and a slower but steady trend toward the acceptance of insects as food, especially among young people and those who adhere to an ecological outlook [29,30,35]. However, long-term processes are needed to ensure the growth of frequent consumption incidents. Governments and other key stakeholders in policy formulation, training institutions, and food producers have the major responsibility of steering change in the delivery of a healthy diet plan. It must be noted that the valuable contributions of DS should concentrate on the safety of food from insects and on campaigns that underline the environmental and health advantages of consuming insects and inspire the creation of appetizing insect-based food products [36,37]

To increase the acceptance and availability of insect-based foods, measures that include proper regulation and good marketing strategies need to be applied. Proper and clear identification, compliance with bold food hygiene standards, and effective communication with clients are essential strategies for enhancing trust and confidence in the products [20,38,39]. Innovation marketing ideas that focus on the green qualities, health value, and convenience of insect-based foods can go a long way toward reducing the level of concern among consumers [20,29,40].

The present research addresses the same subject by examining various factors that affect the acceptance of entomophagy. It was found that the decision to consume insects involves consideration of multiple variables such as demographics and past experience with entomophagy. By considering all of these aspects, the proposed approach aligns with the current research and provides valuable insights into the interconnectedness of these factors and consumer acceptance, as discussed in previous studies [4,9,24].

One significant research finding is that respondents are more accepting of insects as animal feed, particularly those in the younger age group with higher level of education and who have traveled. This suggests an opportunity to encourage people to accept whole insects for human consumption. However, contrasting results were found regarding consumers’ acceptance of hidden insect parts in food items. This highlights the importance of considering the presentation of insect consumption when promoting entomophagy.

Some of the limitations that have impacted this research work include the lack of technology literacy among the majority of the senior citizens targeted in this study. Additionally, there is a common problem of unwillingness among the population to participate in the process for various reasons, such as lack of interest or refusal to provide personal information. It has also been challenging to limit the survey to the territorial council of Castilla y León, as a wider distribution would have resulted in a more diverse sample.

The regional emphasis on the Spanish people in the region of Castilla y León could restrict the generalization of the results to populations other than Spanish individuals that may be prominent in other regions, highlighting the need for large sampling in other populations for further research.

Therefore, future research directions should address these limitations by conducting studies with a larger number of participants from diverse populations. A cross-sectional as well as cross-temporal analysis of consumer’s attitudes towards entomophagy would be informative in advancing the understanding of the acceptance of insects as food. Additionally, focus groups or interviews could provide more detailed insights into psychosocial variables that influence consumer behavior and views on the topic.

From a commercialization standpoint, future research could explore ways to increase the palatability of insects for human consumption, focusing on aspects such as the taste, texture, and packaging appearance of insect products [9,41,42,43]. Furthermore, understanding the nutritional and medicinal values of consuming insects and classifying the compounds that can be derived from insects as drugs could be motivating factors for consumers [13,44].

Therefore, the acceptance of novel insect-based foods by consumers in Western societies depends on various factors, including the consumer perception, culture, and regulations. A better understanding of these aspects is essential when exploring ways to promote the consumption and integration of insect-based foods into Western diets [11,45,46]. However, the findings of the current study suggest that future research should address the limitations of the current study and explore new research avenues to encourage consumer adoption. These challenges must be addressed, and solutions must be implemented to allow entomophagy to thrive and become a successful direction for developing future food sources.

## 5. Conclusions

This study aimed to understand the attitudes and acceptance level of insect-based foods and food products among different age groups in the Castilla y León region, especially among the geriatric population. The results indicated that older adults had a clear negative attitude towards adopting of insects into their conventional meal systems. However, they expressed conditional willingness to consume insects if necessary for survival or if seen as nutritious.

Conversely, younger generations demonstrated a greater propensity for exploring insect-based food options. Prioritizing research efforts to understand their consumption preferences and establish trust in entomophagy products could significantly facilitate their acceptance. Strategically incorporating disguised insect ingredients into familiar food items, improving sensory appeal, and using targeted messaging to highlight the nutritional and ecological advantages of entomophagy could effectively promote market integration.

These findings underscore the need for a multi-faceted approach for unlocking the potential of insects as a sustainable protein source. By implementing such an approach, the groundwork could be laid for routine entomophagy adoption within the Castilla y León population. This requires collaborative efforts from the scientific community, policymakers, and industry representatives to cultivate innovation and promote the culturally sensitive and environmentally responsible adoption of insect-based foods.

## Figures and Tables

**Table 1:** Age distribution.

Age Range (Years)	Percentage of Participants (%)
18–30	27
31–45	22
46–60	27
61–75	18
76 and over 76	6.1

Note: People aged over 76 years accounted for 6.1% of the total sample.

## Data Availability

The original contributions presented in the study are included in the article, further inquiries can be directed to the corresponding author.
